# Outcomes for binge eating disorder in a remote weight-inclusive treatment program: a case report

**DOI:** 10.1186/s40337-023-00804-0

**Published:** 2023-05-22

**Authors:** Caitlin B. Shepherd, Rebecca G. Boswell, Jessica Genet, Wendy Oliver-Pyatt, Christine Stockert, Rebecca Brumm, Shaun Riebl, Elsbeth Crowe

**Affiliations:** 1 Within Health, Coconut Grove, FL USA; 2 Department of Psychology, Smith College, Northampton, MA USA; 3 Princeton Center for Eating Disorders, Penn Medicine, Plainsboro, NJ USA; 4 Department of Psychology, Princeton University, Princeton, NJ USA

**Keywords:** Binge eating, Weight-inclusive, Intuitive eating, Medical complications, Psychiatric comorbidity, Case report

## Abstract

**Background:**

There are no known published reports on outcomes for medically and psychiatrically compromised patients with binge eating disorder (BED) treated remotely in higher level of care settings. This case report presents outcomes of an intentionally remote weight-inclusive partial hospitalization and intensive outpatient program based on Health at Every Size® and intuitive eating principles.

**Case presentation:**

The patient presented with an extensive trauma background and long history of disturbed eating and body image. She was diagnosed with BED along with several comorbidities, most notably major depressive disorder with suicidality and non-insulin dependent diabetes mellitus. She completed a total of 186 days in the comprehensive, multidisciplinary treatment program encompassing individual and group therapy, as well as other supportive services such as meal support and in vivo exposure sessions. Upon discharge, her BED was in remission, her major depressive disorder was in partial remission, and she no longer exhibited signs of suicidality. Overall, she showed decreases in eating disorder, depressive, and anxiety symptoms as well as increases in quality of life and intuitive eating throughout treatment, which were largely maintained after one year.

**Conclusions:**

This case highlights the potential of remote treatment as an option for individuals with BED, especially in cases where access to higher levels of care might be limited. These findings exemplify how a weight-inclusive approach can be effectively applied when working with this population.

**Supplementary Information:**

The online version contains supplementary material available at 10.1186/s40337-023-00804-0.

## Background

Binge-eating disorder (BED) is the most common eating disorder in the United States (US), with an estimated lifetime prevalence of 0.85% and a frequently chronic course of illness (mean duration ~ 16 years) [1]. Individuals with BED often have medical comorbidities including metabolic syndromes like Type 2 Diabetes [1, 2], which may confer increased mortality risk [3]. Although up to two-thirds of individuals with BED live in larger bodies [4], BED poses a unique risk for poor metabolic outcomes beyond that attributable to higher weight status alone [5, 6]. Up to 94% of those with BED also meet diagnostic criteria for another psychiatric disorder, including mood and anxiety disorders and post-traumatic stress disorder (PTSD) [7]. Further, heightened rates of suicide attempts have been found among BED populations [8, 9]. Psychiatric comorbidity has been linked to more severe eating disorder psychopathology and, particularly in the case of mood disorders, decreased likelihood of BED symptoms remitting with treatment [10]. Thus, BED is associated with considerable functional impairment and poor quality of life for sufferers [11, 12] as well as both personal and public health economic burden [13].

Nonetheless, BED often goes undetected, undiagnosed, and untreated. For many individuals with BED, there are significant delays in accessing treatment, including delay estimates of up to 10 years post-symptom onset [14]. A number of factors have been identified that may explain these delays and treatment gaps. First, across eating disorder diagnoses, shame and stigma emerge as common experiences preventing individuals from seeking help [15]. Indeed, low rates of help-seeking behavior have been documented for those with BED, with one study of US adults demonstrating that less than half (49%) ever sought help of any type and only about one-third sought help from a mental-health professional [16]. Furthermore, low levels of public and personal awareness of BED specifically as a distinct and severe eating disorder may interfere [17, 18]. If they do seek treatment, individuals with BED are more likely to present to healthcare facilities for assistance with weight loss or other psychiatric symptoms rather than for an eating disorder [19]. Misconceptions among healthcare professionals about how eating disorder symptoms clinically present may result in under-recognition due to low rates of assessment and diagnostic accuracy, especially for those with BED who live in larger bodies [17, 18]. In fact, in a community sample of US adults, only 3.2% of individuals endorsing symptoms consistent with BED reported receiving a formal diagnosis [20].

Weight-based stigma and discrimination by healthcare providers who ascribe to weight-normative treatment models may impact the quality of care received and contribute to avoidance and decreased treatment utilization for individuals with eating disorders who have higher-weight [17, 21, 22]. Weight-normative narratives, which emphasize weight and weight loss as key determinants of health and well-being, dominate in public health and healthcare arenas [23]. Even within the eating disorder field, disagreement exists regarding the role of weight loss as a treatment goal for those living in larger bodies, with some researchers and providers promoting behavioral weight loss techniques [24]. Data from several meta-analyses show, however, that not only is behavioral weight loss typically associated with short-term, modest weight loss at best but also it appears to be inferior to evidence-based psychotherapeutic interventions in reducing binge-eating symptomatology [25–27]. Moreover, evidence does not support the notion that higher weight or body mass index (BMI) causes health issues nor that losing weight results in improved health [23, 28, 29].

Weight-inclusive practices, which emphasize increasing access to non-stigmatizing healthcare, have been incorporated into recent guidelines for healthcare professionals [28–30]. Weight-inclusive approaches recognize that weight loss is not always a feasible, impactful, or desirable treatment goal and instead focus on improving physical and mental health via health promoting behaviors. Advocates of weight-inclusive practices assert that prescribing weight loss is contraindicated and unethical for those with eating disorders due to the risk of adverse consequences including increased eating disorder psychopathology and weight cycling [23, 29]. The fluctuations of weight cycling may have deleterious effects including increased risk for cardiovascular events, diabetes, and mortality, all of which are already of concern for those with BED [31–33]. Despite concerns expressed by critics that health indicators may not improve in the absence of explicit focus on weight loss, studies have shown improvements in both physical (e.g., blood pressure, blood glucose, cholesterol levels) and psychological (e.g., body image, disordered eating, depression, anxiety, quality of life) domains with non-diet, weight-inclusive treatment approaches, including Health at Every Size® (HAES) and intuitive eating [34–38]. Moreover, a recent systematic review and meta-analysis directly comparing outcomes for weight-inclusive versus weight-loss approaches showed no significant differences between interventions; in other words, these methods were equally effective in terms of improving physical, psychological, and behavioral outcomes [39]. Additionally, weight-inclusive treatment approaches are associated with greater treatment engagement and lower dropout rates [35, 37] and have been reported to decrease shame and negative self-perceptions as well as enhance resiliency in women with BED [22].

In addition to the aforementioned barriers, there is a lack of access to specialized treatment for eating disorders, leading to a “crisis in care” [40, 41]. Given the severity of BED and associated negative consequences when left untreated, there is an urgent need for effective, accessible treatment options. Kazdin and colleagues [41] suggest that technology-based or enabled treatment approaches may help to close this critical treatment gap. For instance, delivering care via telehealth or mobile applications has the potential to increase access by extending the reach of treatment to those who are underserved. Studies have supported the feasibility, acceptability, and efficacy of outpatient eating disorder telehealth treatment [42]. Research has also demonstrated that digital tools (e.g., mobile applications) may be feasible, acceptable, and beneficial adjuncts in the management of eating disorder symptoms [43, 44]. Data collected as a result of the shift to remote treatment during the COVID-19 pandemic illustrate that remote delivered eating disorder treatment is effective for patients in higher level of care settings, including intensive outpatient and partial hospitalization programs [45, 46]. However, these studies have not specifically looked at the benefits according to diagnostic groups, so it remains unknown whether those with BED benefit.

In sum, BED is a common and severe eating disorder that is often undiagnosed and untreated. Shame, low awareness of the disorder, and lack of available care along with the stigma and discrimination inherent in the dominant weight-normative approach to healthcare all serve as barriers to detection and adequate treatment for those with BED. Thus, there is a need for more effective, accessible, and inclusive treatment options, such as technology-enabled programs, to address this crucial gap in care. To our knowledge, there are no studies examining remote treatment at higher levels of care for individuals with BED utilizing a weight-inclusive approach. Hence, we report preliminary outcomes for a patient living in a larger body diagnosed with BED as well as psychiatric and medical comorbidities who was successfully treated in a remote weight-inclusive partial hospitalization program (PHP) and intensive outpatient program (IOP).

## Case presentation

### Treatment program

#### Treatment approach

Within Health is an intentionally remote treatment program offering comprehensive care for patients with eating disorders. The program philosophy is grounded in weight-inclusive practices, incorporating a HAES® framework and intuitive eating principles. The treatment program is integrative and blends evidence-based psychotherapies, including Cognitive Behavioral Therapy (CBT), Dialectical Behavior Therapy (DBT), Acceptance and Commitment Therapy (ACT), with psychoeducation and experiential (e.g., art therapy, movement) modalities. Nutritional rehabilitation in the program relies on Tribole and Resch’s 10 principles of intuitive eating as a foundation [47], which aims to improve disordered eating by extending the concept of mindful eating and emphasizing a shift from external/rule governed eating to internal regulation based on interoceptive awareness. Studies have corroborated the role of interoceptive awareness as a potential treatment mechanism by demonstrating associations with both intuitive eating skills [48–50] and disordered eating [51, 52]. The program’s nutrition approach is also facilitated by use of the Plate-by-Plate® visual, no-numbers approach [53] to meal planning and portioning, which is considered more flexible and intuitive than caloric or exchanged-based meal plans. Drawing from dietetic recommendations as well as the current best evidence for eating disorders, additional nutrition interventions used in the treatment program include education, mindful eating practice, development of practical skills (e.g., meal preparation, grocery shopping), and behavioral strategies (e.g., exposure work, self-monitoring) [54]. A phased approach to nutritional rehabilitation is also utilized based on the level of support needed for renourishment; patients are offered more structure and guidance initially (e.g., logging meals/snacks consumed outside of programming in a mobile application, portioning meals/snacks on camera with staff, receiving meal delivery) and gain freedom and responsibility as they progress through the phases (e.g., decreased frequency of meal logging, portioning independently, selecting snack options). For descriptions of treatment components, see Additional file 2: Table S1.

Services are provided via telehealth by a multidisciplinary team of professionals including a psychotherapist, registered dietitian (RD), registered nurse (RN), psychiatric provider, and clinical support staff (e.g., care partner, food specialist). Patients participate in either the PHP (a minimum of 6 h/day for 5–7 days/week) or IOP (a minimum of 3 h/day for 3–5 days/week) programming which includes individual, couples/family (when warranted), and group therapy along with nutrition counseling, psychiatric intervention, experiential opportunities, and food/meal support. All telehealth services are provided via a mobile application which includes an integrated HIPAA-compliant video conferencing platform. Patient vitals (i.e., weight, blood pressure, heart rate, temperature) are monitored by RNs via remote devices. Grocery and meal deliveries are coordinated by food specialists as needed. The mobile application includes additional features so that patients can access support outside of treatment sessions and groups, including a chat message function, self-guided content, and check-ins (e.g., meal logs). The remote nature of the program enables patients to complete treatment within their home environment and, therefore, aims to increase accessibility of care. Furthermore, while the program was not contracted with any insurance providers at the time of this case report, treatment was covered for the majority of patients by using out-of-network benefits or obtaining single case agreements or gap exceptions, rendering the overall cost to the patient and healthcare system comparable to that of in-person PHP and IOP treatments.

#### Outcome measurement

Patient-reported outcome measures are administered to all patients in the treatment program as part of routine clinical practice to inform treatment planning and monitor progress. Patient-reported outcome measures are completed at admission to capture baseline functioning, monthly during treatment, and then again at discharge. These same measures are administered to patients at 1-, 3-, 6-, and 12-months post-discharge to see how well treatment gains are maintained long-term. The following patient-reported outcome measures are used:

##### Disordered eating attitudes and behaviors

The Eating Disorder Examination Questionnaire (EDE-Q) [55] is a 28-item measure that was used to assess the patients’ disordered eating attitudes and behaviors over the past 28 days. The EDE-Q yields a global mean and four subscale mean scores (i.e., restraint, eating concern, shape concern, and weight concern) reflecting severity of eating disorder symptoms. In addition, it includes data on the frequency of key behaviors, including those relevant for BED (i.e., episodes of overeating, episodes of loss of control eating, days of binge eating).

##### Eating disorder quality of life

The Eating Disorder Quality of Life Questionnaire (EDQOL) [56] is a 25-item measure that was used to assess the patients’ health related quality of life concerns associated with disordered eating over the past 30 days**.** The EDQOL yields a total mean and four subscale mean scores (i.e., psychological, physical/cognitive, work/school, and financial) showing the extent to which key areas of quality of life have been impacted. Notably, higher scores on the EDQOL indicate *lower* quality of life.

##### Intuitive eating

The Intuitive Eating Scale (IES-2) [57] is a 23-item measure that was used to assess the patients’ tendency to engage in practices that are aligned with intuitive eating principles**.** The IES-2 yields a total mean and four subscale mean scores covering various aspects of intuitive eating: unconditional permission to eat, eating for physical rather than emotional reasons, reliance on hunger and satiety cues, and body-food choice congruence.

##### Depressive symptoms

The Patient Health Questionnaire (PHQ-9) [58] is a 9-item measure that was used to assess the severity of the patients’ depressive symptoms. Items for this measure are summed to produce a total score and qualitative descriptors are used to indicate overall severity level.

##### Anxiety symptoms

The State Trait Anxiety Inventory (STAI) [59] is a 40-item measure that was used to assess the patients’ severity of current symptoms of anxiety and degree of anxiety-prone temperament. The STAI yields two subscale scores: state anxiety (i.e., anxiety in the moment) and trait anxiety (i.e., general propensity to feel anxious).

### Patient information

A married, 57-year-old White, female retired US military veteran presented to the treatment program with daily binge eating and night eating symptoms along with depression and trauma symptoms stemming from a history of emotional and sexual abuse. She reported body image issues and weight concerns as well as restrictive eating and dieting behaviors dating back to childhood, with an onset around age eight. She described receiving negative comments about her weight throughout life, including from family and while serving in the military. She indicated having a low sense of self-worth and shame related to her body and eating behaviors, sharing that she has a past and ongoing history of hiding food, eating in secret, and occasionally purging via vomiting when uncomfortably full. She also reported a long history of exercise avoidance, irrespective of pain or injury, related to shame about her body size and physical appearance and awareness of her body during movement. She noted that her symptoms had recently increased in response to the death of her father and resulting conflict with her estranged family members. She identified interpersonal and life stressors, including work and finances, as precipitants for her binge eating behavior.

The patient endorsed current passive suicidal ideation and stated that she had one previous suicide attempt. She denied any prior history of facility-based eating disorder treatment but indicated that she had worked with outpatient providers for psychotherapy and medication management and also had one previous hospitalization for suicidality over ten years ago. Her depressive symptoms were noted to be treatment-refractory despite multiple medication trials. In addition to psychiatric concerns, the patient-reported several relevant medical issues including non-insulin dependent diabetes mellitus, hypercholesterolemia, and irritable bowel syndrome, all of which were well-managed with medication. She also shared that she had gastric bypass surgery approximately 15 years prior. The patient expressed an interest in remote treatment specifically due to having a broken foot which required her to use a wheelchair and made it difficult to leave her house to attend an in-person program. In addition, she had not complied with recommendations to attend a brick-and-mortar program due to shame related to her size and feeling she did not “fit” into the treatment setting.

### Assessment

#### Clinical interviews

Upon admission to the program, the patient met with all members of her multidisciplinary team including a psychiatric provider, RN, RD, psychotherapist, and several clinical support staff for further evaluation. Her initial psychiatric evaluation with a psychiatric nurse practitioner (NP) yielded the following DSM-5 diagnoses: binge eating disorder (BED), major depressive disorder (recurrent, moderate-severe), and PTSD. Mild functional impairment was noted for family and peer relations. Physical health, moderate/severe depression, and affect regulation were identified as symptoms to target. In terms of psychiatric medications, the patient reported that she was currently taking Abilify (2.5 mg daily), Effexor (300 mg daily), Vyvanse (70 mg daily), Ambien (10 mg at bedtime), and Xanax (0.5 mg as needed).

Her RN conducted an initial nursing assessment, including administering the Lifetime/Recent version of the Columbia Suicide Severity Rating Scale [60]. The patient endorsed recent (i.e., past month) suicidal ideation, with a severity level of 3 (i.e., active suicidal ideation with method but without plan or intent to act) and an intensity of 11, placing her in the moderately severe range (i.e., 11-5). She denied any recent suicidal behavior. She reported recent and longstanding physical pain in her neck, shoulders, and back that interfered with her life, rating it a 2 to 3/10 when she took pain relieving medications (i.e., NSAIDs, acetaminophen) and a 7/10 when she was unmedicated. Her recent labwork (completed 5 days prior to admission) was reviewed and showed an elevated hemoglobin A1C level (i.e., 6.3%) in what is considered the prediabetic range (normal value is below 5.7%; prediabetic range is between 5.7 and 6.4%, diabetic level is 6.5% and above), which is consistent with her known non-insulin dependent diabetes mellitus diagnosis. In addition, her total cholesterol (i.e., 148 mg/dl; normal value is below 200 mg/dl) and HDL (i.e., 47 mg/dl; normal value is between 35 and 80 mg/dl for women) were both in the normal range. Her vital signs (i.e., sitting and standing heart rates and blood pressures) were all within the normal range with no signs of cardiovascular complications.

The patient’s initial nutrition evaluation with her RD revealed a pattern of daytime food restriction and subsequent evening binge eating and night eating. She described restricting her overall intake during the day, for instance by skipping meals and counting calories/macronutrients, as well as the variety of foods consumed, attempting to stick to safe “nutritious foods” and refrain from eating “fattening” foods. Based on evaluation of a 24-h dietary recall, her RD estimated that the patient was typically consuming excessive calories (i.e., estimated energy needs plus ~50–100% of overall estimated energy needs) as a result of her binge and night eating behavior as well as excessive caffeine and diet beverages (i.e., approximately 3 1/2 litres of caffeinated diet soda per day). For diagnostic clarity, both her RD and psychotherapist inquired about the size and affective reaction during binge eating episodes. She described a typical binge as starting with eating a “large portion for dinner” (e.g., chicken nuggets) around 5:30 pm and then continuing to eat (e.g., sunflower seeds, ice cream bar, peanut butter cups, bagel with butter) until bedtime around 8:30 pm, beyond comfortable fullness. She noted that she cannot eat much in a short period of time because of her gastric bypass surgery, so her binges last for several hours throughout the evening. She reported feeling “sad”, “lonely”, and “anxious” during her binge episodes and experiencing “minimal control”. In addition to these discrete binge episodes, the patient reported waking up multiple times per night and then feeling like she “has to eat something because her mouth is dry”. She described eating “handfuls of food” (e.g., sunflower seeds, candy) before going back to sleep. The psychotherapist confirmed that the patient met criteria for BED with concurrent night eating behaviors. The patient also shared that she was currently unable to exercise due to her broken foot but stated that when she exercised in the past, she “didn’t enjoy it” and “avoid[s] movement”.

The treatment team identified a number of psychosocial stressors and risk factors for the patient including a history of sexual abuse and trauma, a recent loss in the family, a history of suicidal behavior, and a lack of social support from friends and family. They also identified several notable strengths including the patient’s self-sufficiency, assertiveness, tenacity, and resilience. Her psychotherapist noted that she was cooperative and had fair insight/judgment regarding her condition and treatment needs. The patient stated that she anticipated that treatment would “bring up a lot of [her] issues” and be “painful” but that she was hopeful that she would lose weight with “better control over [her] behaviors”. The team documented that she was amenable to the treatment plan and motivated for recovery.

#### Baseline outcome measures

The patient’s scores on baseline patient-reported outcome measures overall showed a high level of disordered eating attitudes and behaviors that were substantially impacting her quality of life, a lack of intuitive eating skills, and heightened mental health symptoms (see Table 1 for complete listing of baseline scores, clinical cutoff/norm values, and comparisons). At baseline, her global EDE-Q was between the 60–70th percentile for women with BED [61]. In addition, her global EDE-Q and subscale scores were within a standard deviation of mean scores reported by patients with BED in prior research [62] and are thus consistent with expectations for this population. She reported 15 episodes of binge eating (i.e., eating an unusually large amount of food and experiencing a sense of lost control) over the last 28 days for an average of 3.75 binge eating episodes per week, indicating moderate illness severity. Her EDQOL total and subscale scores were also in line with mean levels previously reported for a clinical sample with moderate eating disorder symptoms, demonstrating that her quality of life was being affected by her eating disorder [56]. Due to an oversight, the patient did not complete the IES-2 at admission; thus, her first IES-2 total and subscale scores obtained at one month of treatment were within the range expected based on the mean reported for individuals with BED [63]. Her depression score on the PHQ-9 indicated moderately severe depression [58]. Finally, her trait anxiety score on the STAI was above the cutoff for clinically significant anxiety [64] while her state anxiety was just below the cutoff.

**Table 1:** Baseline, discharge, and 12-months post-discharge scores across patient-reported outcome measures

Measure	Subscale	Cutoffs/norms	Baseline	Discharge	12-Months post-discharge	Δ During treatment	Δ 12-Months post-discharge
EDE-Q		3.89 (1.04)	3.88^a^	1.44	0.94	− 2.44^b^	− 0.50
	Restraint	2.43 (1.67)	3.60^a^	0.20	0.40	− 3.40	+ 0.20
	Eating Concern	3.45 (1.34)	3.40^a^	0.40	0.20	− 3.00	− 0.20
	Shape Concern	4.91 (1.15)	4.50^a^	2.75	1.75	− 1.75	− 1.00
	Weight Concern	4.18 (1.12)	4.00^a^	2.40	1.40	− 1.60	− 1.00
EDQOL		1.29 (0.54)	1.64^a^	0.32	0.28	− 1.32	− 0.04
	Psychological	2.20 (0.89)	2.78^a^	0.89	0.56	− 1.89	− 0.33
	Physical/Cognitive	1.52 (0.76)	1.83^a^	0.00	0.33	− 1.83	0.33
	Financial	0.38 (0.69)	0.80^a^	0.00	0.00	− 0.80	None
	Work/School	0.24 (0.54)	0.20^a^	0.00	0.00	− 0.20	None
IES-2		2.18 (0.42)	1.91^a^	3.57	3.61	+ 1.66	+ 0.04
	UPE	2.87 (0.75)	2.50^a^	4.50	4.00	+ 2.00	− 0.50
	EPR	1.68 (0.68)	1.25^a^	3.63	4.00	+ 2.38	+ 0.27
	RHSC	1.88 (0.67)	2.17^a^	3.17	3.67	+ 1.00	+ 0.50
	BFCC	2.51 (0.93)	2.00^a^	2.33^a^	1.67^a^	+ 0.33	− 0.66
PHQ-9		4	19^a^	5^a^	4	− 14^b^	− 1
STAI-S		40	39	21	21	− 18^b^	None
STAI-T		40	61^a^	33	36	− 28^b^	+ 3

*EDE-Q* Eating Disorder Examination Questionnaire, *EDQOL* Eating Disorder Quality of Life Questionnaire, *IES-2* Intuitive Eating Scale, *UPE* Unconditional Permission to Eat subscale, *EPR* Eating for Physical Reasons subscale, *RHSC* Reliance on Hunger and Satiety Cues subscale, *BFCC* Body-Food Choice Congruence, *PHQ-9* Patient Health Questionnaire, *STAI-S* State Trait Anxiety Inventory-State, *STAI-T* State Trait Anxiety Inventory-Trait

^a^Score was in clinical range based on reported clinical cutoff and norm values

^b^Magnitude of change in patient’s scores from baseline to discharge represents a clinically significant treatment response

### Therapeutic intervention

Based on her initial evaluations and assessments, it was determined that the patient initially met criteria for PHP level of care. More specifically, the following factors indicated that the American Psychiatric Association (APA) level of care guidelines for PHP level of care were met [65]: spending 50–75% of the day thinking about food/weight/body image (i.e., being preoccupied with intrusive, repetitive thoughts > 3 h/day), having co-occurring depression and PTSD requiring management, needing some structure to acquire, prepare, and consume food properly, and having limited support and structure in her environment. She was deemed appropriate for 5 days per week of programming. Her recommended treatment plan included individual and group therapy components as well as additional supportive services, as shown in Table 2. She was prescribed a maintenance meal plan of 3 meals and 3 snacks per day to help normalize her eating patterns and break the restrict-binge cycle (i.e., a pattern whereby individuals restrict their eating, often in an effort to lose weight and/or change their body shape, end up overeating or bingeing due to deprivation, and then resume restricting to compensate). The intuitive eating-based nutrition counseling approach, overseen by the patient’s RD and reinforced by clinical support staff), was tailored to account for the patient’s medical conditions (i.e., non-insulin dependent diabetes mellitus, irritable bowel syndrome). For instance, in line with the intuitive eating principle of honoring your health, she was encouraged to pay attention to how certain foods influenced her emotional/physical states and was provided with education about macronutrient metabolism and strategies to maintain blood glucose levels to help guide her choices. The patient’s overall treatment goals and objectives at the beginning of treatment are detailed in Table 3.

**Table 2:** Multidisciplinary treatment plan

Individual	Group	Support
Individual psychotherapy (psychotherapist)	*Psychoeducational* Nutrition Physiology	*Care Partner* Meal/snack Milieu ADL
Nutrition counseling (RD)		
Psychiatric evaluation/management (psychiatric NP)	*Psychotherapeutic Skills* Positive Psychology ACT CBT DBT Process Body Image Family and Relationships	*Nurse Monitoring* Medication Labs
Nursing follow-up care (RN)		
Support sessions/experiential opportunities (care partner)		
Couples/family therapy (as needed with psychotherapist)		
	*Experiential* Art Therapy Autobiography Cooking Psychodrama Movement Breathwork	
	*Support* Journal Sharing Self-Care Treatment Successes	

*Note.* PHP and IOP levels of care included the same elements but differed in terms of frequency and intensity of engagement in components of treatment. See Additional file 1: Figure S1 for a detailed breakdown of the patient’s treatment timeline and Additional file 2: Table S1 for descriptions of treatment components.

**Table 3:** Treatment goals and progress

Goal	Objectives	30-Day	60-Day	90-Day	120-Day	150-Day progress	Discharge progress
1. Decrease the frequency and intensity of daily bingeing and night eating episodes and increase daytime and overall nourishment *Client’s words:* To “not be obsessed by food”, “have a normal relationship with food”, and “cook more fresh foods”	A. Eliminate daily bingeing and night eating episodes	Bingeing less than 1×/day; Night eating occurring 1×/night	Bingeing 1-2×/week; Night eating occurring less than 1×/night	No longer bingeing; Night eating occurring more than 1×/night	Night eating occurring during 1 out of 3 wake periods	Night eating occurring 1×/night	Remains active
	B. Eat according to meal plan routinely	Improvement but lacking consistency	Accomplished at home; Applying outside of the home	Completing meal plan but having urges to restrict	Missed some meals and snacks when not at home	Met	Continues to complete 100% of meal plan
	C. Practice mindful and intuitive eating skills daily	Recognizes fullness and completes daily meal logs	Able to honor fullness	Working on feeling hunger cues	Recognizes hunger and engages in distraction- free eating	Practicing mindful eating	Remains active
	D. Prepare/cook and try new foods several times a week	Cooking at least 1×/week in cooking class and trying new foods more than 2×/week	Tried additional new foods and recipes	Cooking meals and trying new foods at least 2×/week	Cooking and trying new foods independent of cooking class	Met	Continues to regularly prepare/ cook and try new foods
2. Examine and mend her connection to her body and adopt caring thoughts about her body *Client’s words:* To “move more in general” and “sit less and get out of the house more”	A. Engage in gentle movement to increase strength and flexibility several times a week	Attending movement group 2×/week	Not attending movement group consistently; Moving more in daily activities	Attending movement group when available; Completing household chores	Attending movement group; Going for walks with care partner	Met	Attending fitness program up to 3×/week outside of program
	B. Experience joy in movement several times a week	Participating but not experiencing joy	Exploring types of movement that bring her joy	Creating list of dance songs and having a “dance party” at home 1×/week	Enjoying walks with care partner; Reports enjoying movement group 50% of the time	Continue to enjoy walks; Appreciates movement group more with music she likes	Remains active
	C. Challenge negative body image thoughts and develop sense of self outside of body appearance	Reminding herself that others like her for who she is not what she looks like	Recognizing sources of beliefs about beauty and weight	Struggling with increase in negative body image thoughts	Challenging belief that positive sense of self is tied to weight	Shifting focus to other aspects of self when having negative body image thoughts	Remains active
3. Improve general sense of self-worth and challenge negative thoughts about herself *Client’s words:* To “be kinder” and more “compassionate to myself”	A. Replace negative self-talk with positive or neutral self-talk	Using affirmations daily	Struggling with increase in negative self-talk	Refraining from family interactions that trigger negative self-talk	Negative self-talk has decreased	Able to rephrase negative self-talk into more neutral statements	Remains active
	B. Explore core beliefs underlying negative self-talk and challenge	Recognizing core beliefs about being a “bad person”	Practicing challenging core belief that she is “unworthy”	Able to accept that she is “worthy”	Experiencing greater sense of self-respect	Realizing that core beliefs are not accurate	Remains active
	C. Engage in self-care and fulfilling activities several times a week	Engaging in self-care (e.g., doing hair and makeup) 2×/week; Attending art class and spending time with pets 2×/week	Met	Unable to attend class class but spending more time with spouse	Met		Continues to engage in self-care and participate in fulfilling activities
4. Decrease depressive symptoms, PTSD symptoms, and process past trauma *Client’s words:* To “be more mindful” and “listen more”	A. Practice mindfulness and emotion regulation daily	Practicing mindfulness daily during meals/snacks	Experiencing increased comfort with mindfulness	Using relaxation skills and working on regulating emotions by ensuring adequate sleep	Continuing to use tools daily to promote mindfulness and emotion regulation	Sharing progress with others in group	Remains active
	B. Gain greater insight into the emotional states of self and others	Developing awareness of others’ perspectives; Receiving compliments without assuming malintent	Able to understand perspective of others with greater ease	Working on identifying and understanding own emotional state	Met		Continues to recognize emotional states of self and others
	C. Report and monitor any SI and commit to the prepared safety plan	Denies any SI since start of program	Fleeting passive SI but no plan; Committing to safety plan	No SI in over 2 weeks	No SI for 4-6 weeks	No SI for 2-3 months	Continues to be free from SI

### Follow-up and outcomes

#### Outcomes

**Fig. 1:**
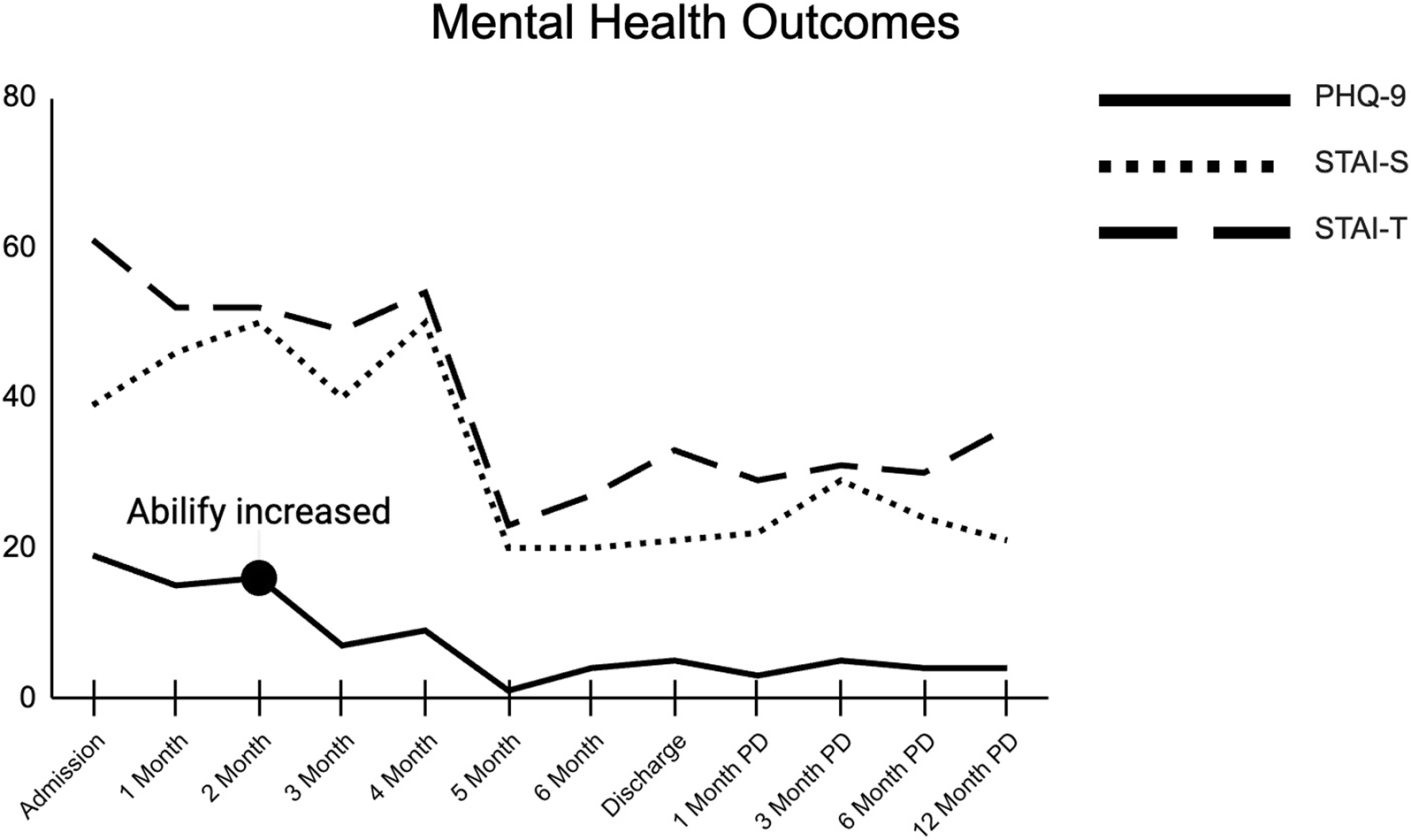
Change in mental health symptoms throughout treatment and post-discharge

Overall, the patient completed 15 weeks at the PHP level of care and then stepped down to the IOP level for 11.5 weeks (see Additional file 1: Figure S1 for a detailed diagram of the patient’s treatment timeline). She started IOP at 5 days per week and tapered down to 3 by discharge. In addition to the individual sessions outlined in Table 2, the patient had intermittent couples/family therapy sessions with her spouse. She showed good insight/judgment, was engaged and cooperative throughout treatment, and generally adhered to her treatment plan. While in PHP, she did initially struggle to attend movement groups due to exercise avoidance; however, after having several individual meetings with the group facilitator she felt more comfortable and was able to participate in the group sessions. After 2 months of treatment, the patient’s dosage of Abilify was increased to 5 mg daily, which was observed to have a mood stabilizing effect. This medication change also coincided with a precipitous decrease in reported depressive symptoms; her PHQ-9 score decreased by 9 points from 16 points at 2 months to 7 points at 3 months (see Fig. 1). Throughout treatment, the patient was also titrated off Ambien but continued to take Xanax at bedtime (as needed) and added melatonin as a sleep aid.

After 186 days, the patient was discharged from the treatment program. At the time of her discharge, she no longer met criteria for BED as she had refrained from engaging in binge eating behavior for approximately 2 months and her diagnosis of MDD was noted to be “in partial remission” with no current suicidal ideation. The patient had met the majority of her treatment goals, as detailed in Table 3. More specifically, she was following her meal plan, preparing and trying new foods with regularity, attending a fitness program, engaging in self-care and fulfilling activities, and recognizing emotional states of herself and others. Her remaining active goals were deemed appropriate for outpatient level of care, including the following: eliminating remaining night eating behaviors (i.e., 1x/night), practicing mindful and intuitive eating skills, engaging in joyful movement, improving body image and sense of self-worth, and building emotion regulation skills. The patient’s vital signs (i.e., heart rate, blood pressure) remained stable throughout treatment. Although remaining in the range expected for someone with non-insulin dependent diabetes mellitus, her hemoglobin A1C level did increase from 6.3% at intake to 6.9% at discharge, likely due to her increased Abilify dosage [66]. Her total cholesterol (i.e., 171 mg/dl) and HDL (i.e., 50 mg/dl) remained within the normal range. By the end of treatment, she reported no pain.

The patient was discharged with the recommendation that she continue treatment with her multidisciplinary outpatient team consisting of a psychotherapist, psychiatric provider, and primary care provider. It was recommended that her primary care provider continue monitoring her hemoglobin A1C level moving forward to ensure that no medication adjustments or additional interventions were needed. In addition, it was recommended that she obtain an outpatient RD to see on a weekly basis to maintain and continue her progress. She was also encouraged to consider trauma-specific treatment such as eye movement desensitization and reprocessing. Lastly, she was encouraged to attend support groups several times per week.

##### Discharge outcome measures

**Fig. 2:**
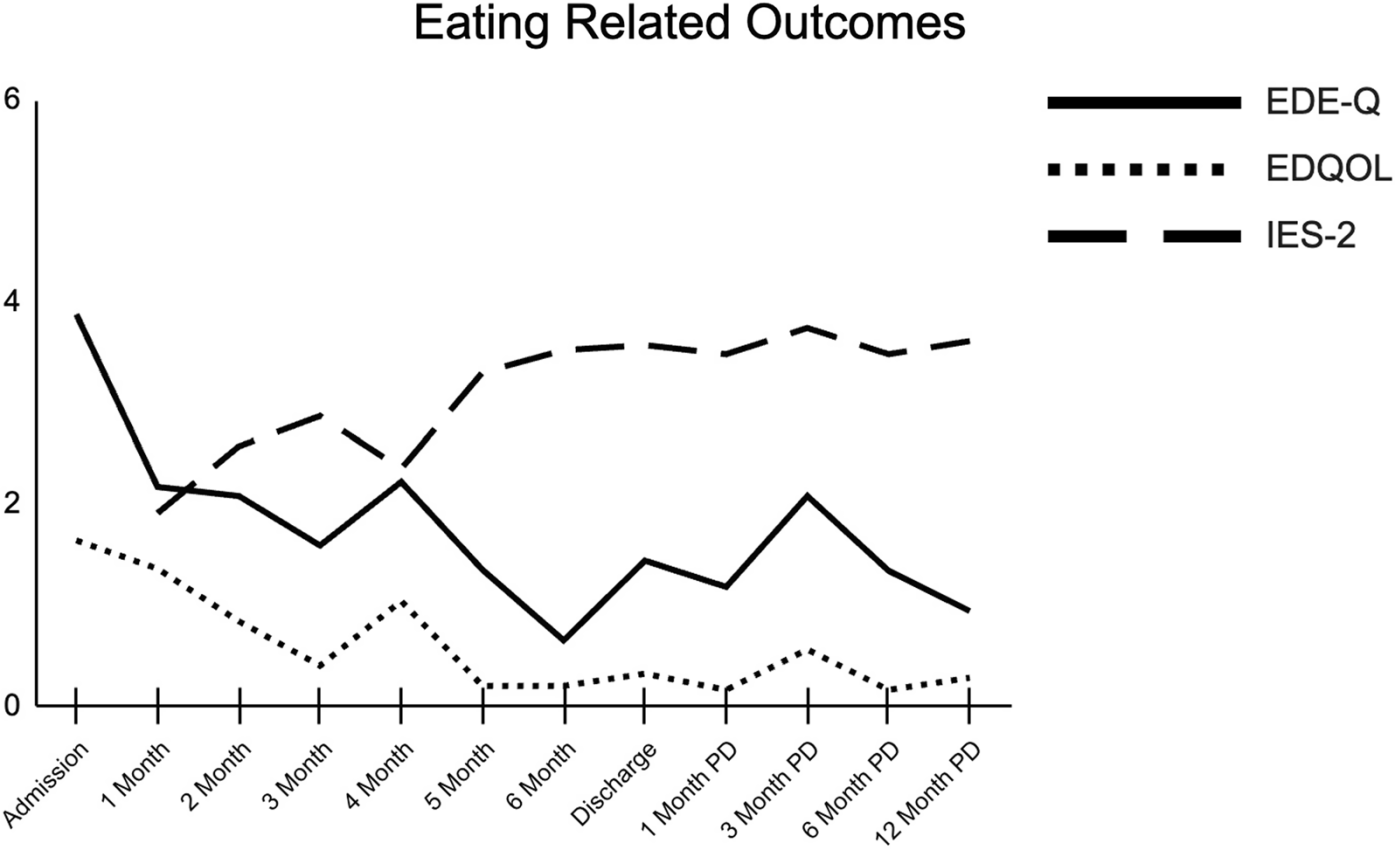
Change in eating related outcomes throughout treatment and post-discharge

**Fig. 3:**
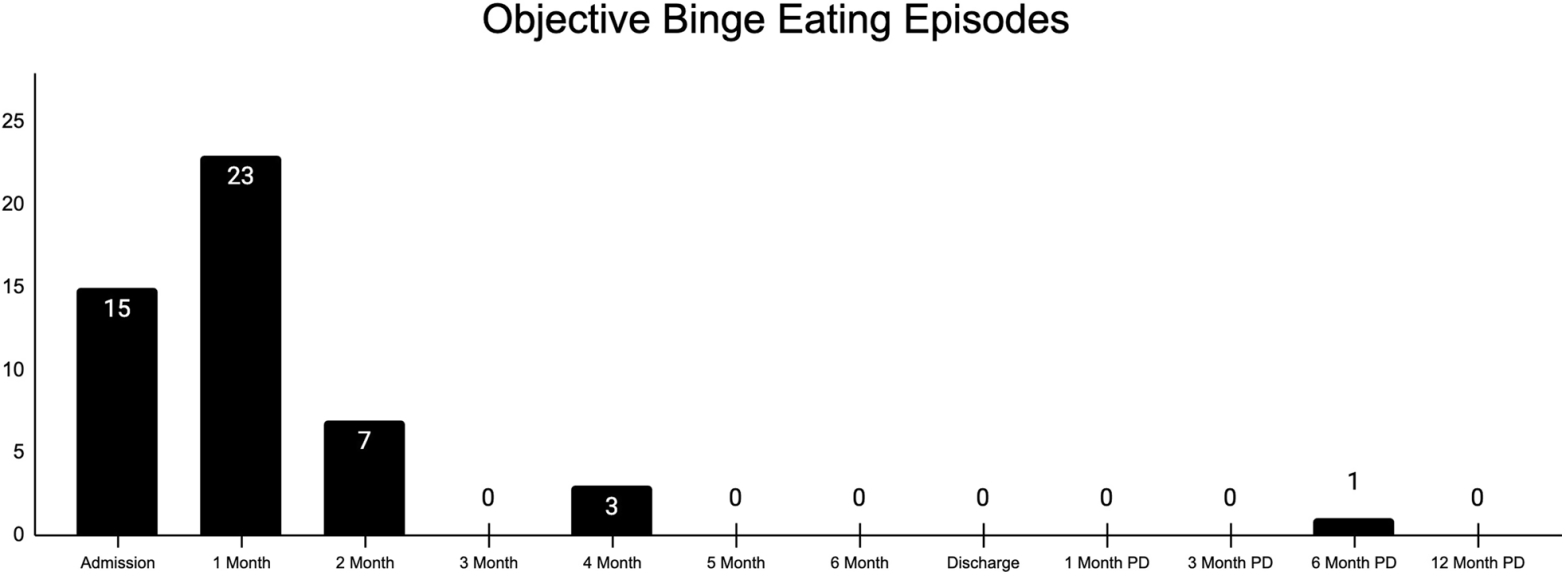
Change in frequency of objective binge eating episodes throughout treatment and post-discharge

At discharge, the patient’s scores on patient-reported outcome measures were indicative of eating disorder remission and improvement in mental health (see Figs. 1, 2, and 3 for graphs of all data collection points during treatment; see Table 1 for complete listing of discharge scores and magnitude of change during treatment). Her global EDE-Q score dropped to the 5th percentile for women with BED [61] and she reported no objective episodes of binge eating over the last 28 days by the end of treatment. At discharge, all of her EDE-Q scores were more than a standard deviation below reported means for patients with BED [62], demonstrating that her eating disorder symptoms were no longer in this clinical range. The magnitude of change in her global EDE-Q signifies a reliable and clinically significant treatment response based on prior studies of clinical samples [67, 68]. The patient’s quality of life also improved by discharge, as evidenced by decreases in her EDQOL scores to levels consistent with means reported for those without an eating disorder [56]. Additionally, the patient’s intuitive eating skills improved by discharge; her total IES-2 as well as her unconditional permission to eat, eating for physical rather than emotional reasons, and reliance on hunger and satiety cues subscale scores were all more than a standard deviation above the mean reported for individuals with BED [63]. Only her score on the body-food choice congruence subscale remained within the range for those with BED. The patient’s depressive symptoms on the PHQ-9 decreased to the mild range [58], indicating a clinically significant change (i.e., more than 5 points) as well as a full treatment response (i.e., at least 50% reduction) and near remission of symptoms (i.e., scores < 5). She experienced a clinically significant change in both her state and trait anxiety as measured by the STAI (i.e., more than 10 points). Additionally, both her state and trait anxiety were below the cutoff for clinically significant anxiety [64] at discharge.

#### Follow-up

After discharge, the patient elected to take advantage of alumni services provided by Within Health. She attended approximately 2–3 free weekly support group sessions per month (a total of 31 times) over the course of one year following her discharge. She also opted to continue working with clinical support staff (i.e., care partner) for additional support and attended 12 sessions for a fee during that time period.

##### Post-discharge outcome measures

The patient completed all post-discharge outcome measures (see Figs. 1 and 2 for graphs of all post-discharge data collection points). Overall, her scores suggest that gains were maintained over time, up to one-year after treatment (see Table 1 for complete listing of scores and magnitude of change at 12-months post-discharge). By 12-months post-discharge, EDE-Q, EDQOL, and IES-2 scores all remained at levels comparable to her discharge scores. She reported one objective episode of binge eating over the last 28 days at 6-months post-discharge but none at other time points. Her depressive symptoms dropped to 4, indicating a remission of symptoms (i.e., scores < 5). Her state and trait anxiety remained relatively stable below the cutoff for clinical significance.

### Patient perspective

The patient was asked if she wanted to provide a brief statement about her experience of treatment. She shared the following approximately 14 months after she discharged from the program.

"I have an eating disorder with psychological issues. It was the best medical experience to date. This program treats the whole person. I felt truly cared for. The experience was like being enveloped in a big hug. The staff was amazing. Every issue was addressed. I no longer binge. They gave me the tools and care I needed to break the cycle."

## Discussion and conclusions

This case report illustrates how a remote weight-inclusive treatment program can be a feasible and beneficial option for individuals with BED who require the structure and stability of a higher level of care setting. As evidence of the potential effectiveness of the program, the patient was able to normalize her eating patterns, eliminate binge eating episodes, and decrease night eating symptoms such that she no longer met criteria for an eating disorder after completing the program. In addition, she was experiencing only mild symptoms of comorbid depression and showed decreased symptoms of anxiety at discharge. She also showed substantial progress towards her treatment goals as well as improved scores on patient-reported outcome measures, which were largely maintained at one-year follow-up. The clinical outcomes for this patient are especially remarkable given that she had struggled with disordered eating symptoms for nearly 50 years. The ability to engage in treatment from home appeared to be helpful for this patient, especially early in her treatment when she was experiencing mobility issues related to her broken foot. The flexibility and convenience afforded by remote treatment may have contributed to successful outcomes in this case too as the patient demonstrated high engagement and adherence. Given the patient’s reported shame with respect to her weight and appearance, the weight-inclusive approach employed by this treatment program was likely an asset. The patient presented in this case report demonstrated a fairly typical profile for BED: chronic course [1], common psychiatric and medical comorbidities [1, 2, 7], a history of suicidal ideation and behavior [8, 9], functional impairment and decreased quality of life [11, 12], and treatment delay [14]. Hence, this remote weight-inclusive approach shows promise as a way of increasing access to specialized eating disorder treatment for others suffering from BED. Since prior research has suggested that PHPs in particular are an essential part of the eating disorder treatment continuum, with savings estimated at approximately $9,645 per patient compared to inpatient care [69], this treatment program also represents a cost-effective option.

There are several notable implications of this case report of a patient with BED receiving remote higher level of care, weight inclusive eating disorder treatment. First, this initial case report adds to the literature showing that remote delivery of higher level of care eating disorder treatment may be feasible, acceptable, and effective [42–46] by specifically demonstrating a benefit for an individual with BED. This report also suggests that this delivery has the potential to increase access to treatment for individuals who (a) have limited mobility and/or (b) experience internalized shame/stigma related to their condition that limits treatment engagement and/or (c) experience other psychosocial barriers to engaging in brick-and-mortar treatment. Future work should continue to explore this treatment modality as a way to improve access to evidence-based eating disorder care [41]. Second, this report highlights effective weight-inclusive treatment practices, which resulted in significant reduction in eating disorder and comorbid psychopathology without adverse health consequences, consistent with prior research findings [34–39]. Additional research should continue to evaluate the efficacy of using weight-inclusive practices in remote higher level of care settings and, if similar results are confirmed using more robust methodologies, explicitly incorporate recommendations into existing guidelines for eating disorder care [28–30]. Together, this work provides an early suggestion that remote, weight-inclusive higher level of care treatment for eating disorders could improve access to and outcomes of eating disorder treatment, especially for individuals with BED who may otherwise not have access to care.

This case report should be understood in the context of its limitations. It is unknown what components of the treatment program account for the positive outcomes experienced by this patient and to what extent the remote and weight-inclusive approach can be credited. Future research should seek to directly compare outcomes to those obtained in programs based on other treatment models, as well as examine within-treatment measures that can highlight mediators and moderators of treatment outcome in a larger sample of patients. Additionally, quantitative and qualitative data could be gathered from patients regarding the perceived benefits of treatment and changes in hypothesized mediators including internalized weight-stigma or anti-fat bias. Furthermore, while this case synthesized multiple sources of information to draw conclusions, including validated patient-reported outcome measures, clinical interviews/evaluations, and treatment plan updates, the data collected were not exhaustive. Other patient-reported outcome measures, for instance those measuring PTSD symptoms (e.g., PTSD Checklist for DSM-5) [70] and night eating behaviors (e.g., Night Eating Questionnaire) [71], as well as additional objective data points (e.g., blood glucose level, mobile application engagement metrics) could provide a more comprehensive and nuanced picture of the treatment program’s impact on psychological and physical health.

The patient presented in this report demonstrated several advantages and strengths that made her a good candidate for this treatment program, thereby limiting the generalizability of results to other individuals with BED who do meet these criteria. She had the necessary resources to participate in the treatment program, including access to the internet and a smartphone. She also had insurance with out-of-network benefits that covered the cost of the treatment program; as a result, she was able to remain in treatment until stepping-down to the outpatient level of care was clinically indicated. Financial issues and inadequate/lack of insurance coverage are frequently-cited barriers for individuals with eating disorders [15, 72]. Finally, the patient showed fair to good insight/judgment regarding her condition and was highly motivated for treatment. Both denial/failure to perceive the severity of one’s illness and low motivation for change commonly function as barriers to help-seeking for those with eating disorders; therefore, in this regard, the patient in this report may be less typical [15, 72].

Nevertheless, this case report is the first to note the effectiveness of a remote higher level of care treatment program for eating disorders, demonstrating a specific benefit for BED. This report illustrates how such a program can incorporate weight-inclusive principles by setting treatment goals focused on symptom reduction, behavior change, and improved quality of life without an emphasis on weight loss. While further research is needed to establish the efficacy of this approach and elucidate treatment mechanisms, this case report suggests a promising avenue for increasing the accessibility of non-stigmatizing treatments for BED.

## Supplementary Information

**Additional file 1**: **Figure S1**. Diagram of the Patient’s Treatment Timeline.

**Additional file 2**: **Table S1. **Descriptions of Treatment Components.

## Data Availability

All data generated or analyzed during this study are included in this published article.

## Abbreviations

BED: Binge eating disorder
US: United States
HAES: Health at Every Size®
PTSD: Post-traumatic stress disorder
BMI: Body mass index
PHP: Partial hospitalization program
IOP: Intensive outpatient program
RD: Registered dietitian
RN: Registered nurse
HIPAA: Health Insurance Portability and Accountability Act
EDEQ: Eating Disorder Examination Questionnaire
EDQOL: Eating Disorder Quality of Life Questionnaire
IES-2: Intuitive Eating Scale
PHQ-9: Patient Health Questionnaire
STAI: State Trait Anxiety Inventory
NP: Nurse practitioner
DSM-5: Diagnostic and Statistical Manual of Mental Disorders
