# Supplementary figures and images for: Outcomes for binge eating disorder in a remote weight-inclusive treatment program: a case report
Source: J Eat Disord. 2023 May 22;11:80. doi: 10.1186/s40337-023-00804-0 (PMC10201521; doi:10.1186/s40337-023-00804-0)

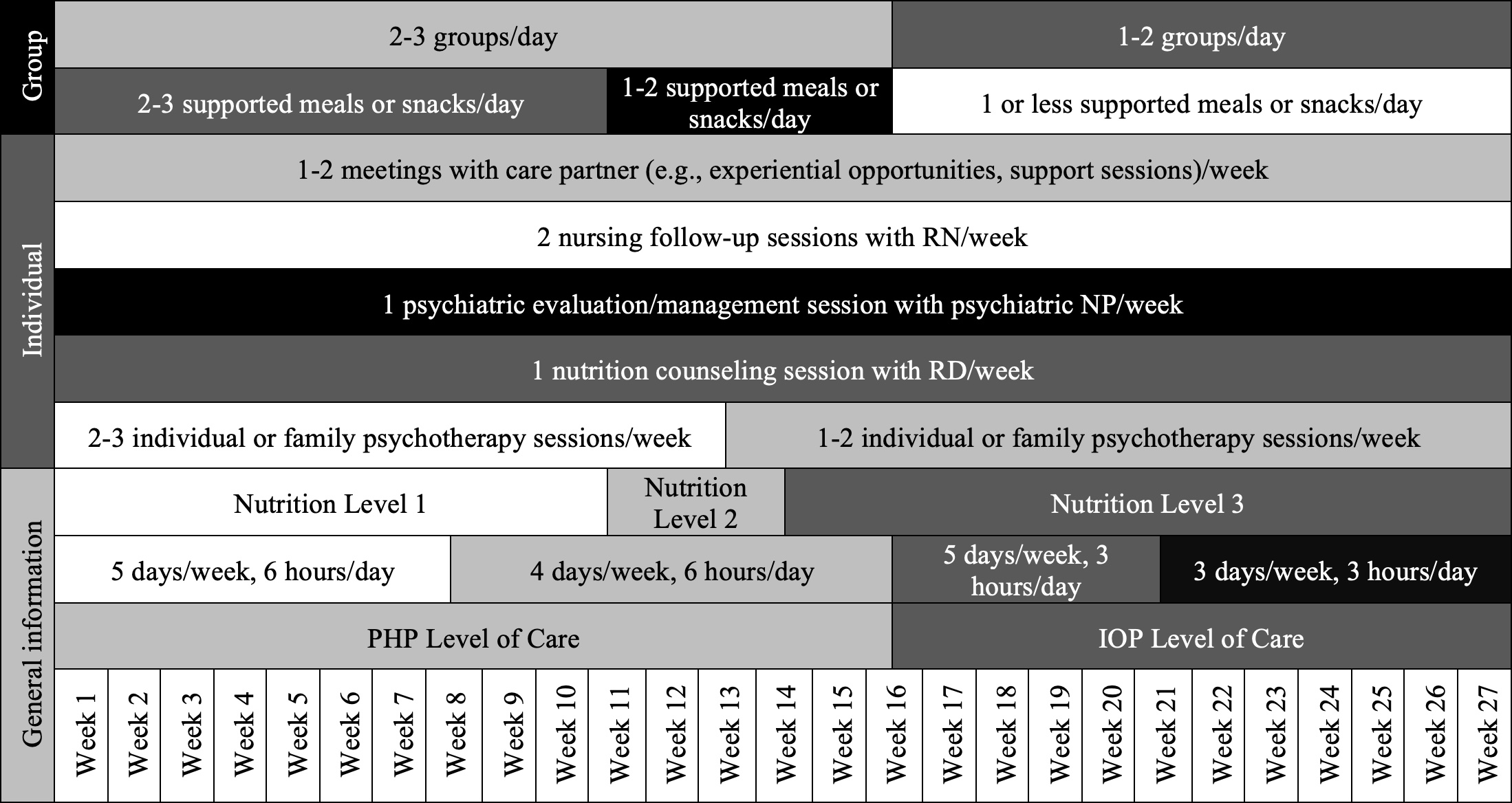

Supplement: Supplementary file 1 — Additional file 1: Figure S1. Diagram of the Patient’s Treatment Timeline. [file 40337_2023_804_MOESM1_ESM.jpg]
