# Supplementary material for: Outcomes for binge eating disorder in a remote weight-inclusive treatment program: a case report
Source: J Eat Disord. 2023 May 22;11:80. doi: 10.1186/s40337-023-00804-0 (PMC10201521; doi:10.1186/s40337-023-00804-0)
Supplement: Supplementary file 2 — Additional file 2: Table S1. Descriptions of Treatment Components. [file 40337_2023_804_MOESM2_ESM.docx]

**Table S1. Descriptions of Treatment Components**

| **Individual Components of Treatment** | |
| --- | --- |
| Individual psychotherapy | 45-minute session with psychotherapist using various therapeutic techniques as indicated based on patient presentation (e.g., ACT, CBT, DBT, interpersonal) |
| Nutrition counseling | 45-minute session with RD to review progress, set goals, learn about nutrition, and gain support |
| Psychiatric evaluation/management | 15-minute session with psychiatric provider to review symptoms and medication issues |
| Nursing follow-up care | 15-minute session with RN to obtain vitals and assess medical status |
| Support sessions/experiential opportunities | 45-minute session with care partner to provide support and/or engage in experiential opportunities (e.g., in-vivo food or body-related exposures) |
| Couples/family therapy | 45-minute session with psychotherapist to address couples/family-related issues including increasing understanding of eating disorders, working on communication, and cultivating supportive relationships |
| **Group Components of Treatment** | |
| Nutrition | Psychoeducational group providing information about nutrition to help patients challenge disordered food and eating beliefs. |
| Physiology | Psychoeducational group providing information about how bodies typically work and how eating disorders may impact physiological functioning. |
| Positive Psychology | Skills group that encourages self-compassion through the use of Positive Psychology techniques. |
| ACT | Skills group designed to increase psychological flexibility through ACT principles including practicing mindfulness and engaging in values-based behaviors. |
| CBT | Skills group that teaches CBT techniques including cognitive restructuring and problem solving to manage eating disorder thoughts and urges. |
| DBT | Skills group covering the four DBT modules: core mindfulness, distress tolerance, emotion regulation, and interpersonal effectiveness. |
| Process | Unstructured group where patients are able to give and receive feedback, develop self-awareness, practice social skills, and connect with others in a supportive, non-judgmental environment. |
| Body Image | Group focused on fostering body appreciation and acceptance via multiple therapeutic approaches (e.g., ACT, strengths-based, mindfulness). |
| Family and Relationships | Group centered around improving interpersonal patterns and gaining insight into relationship dynamics. |
| Art Therapy | Experiential group using art as a medium to explore and externalize thoughts and emotional experiences. |
| Autobiography | Experiential group incorporating autobiographical writing and personal storytelling. |
| Cooking | Experiential group where food specialists demonstrate how to prepare different foods and guide patients through recipes. |
| Psychodrama | Experiential group in which patients use dramatization to process conflicts and issues related to their eating disorder. |
| Movement | Experiential group offering different movement modalities including yoga and strength training. |
| Breathwork | Experiential group using mindfulness and guided breathwork to improve physical and mental health. |
| Journal Sharing | Support group where patients reflect on and receive encouragement around journaling activities and exposure work. |
| Self-Care | Support group emphasizing engagement in self-care activities to promote positive well-being. |
| Treatment Successes | Support group in which patients set personal treatment goals, challenge one another to grow, and celebrate progress. |
| **Support Components of Treatment** | |
| Meal/Snack | Care partners assist patients with meal/snack portioning, pre meal/snack check-ins, and post meal/snack processing |
| Milieu | Care partners offer emotional support as needed outside of programming hours (e.g., via mobile app chat function) |
| Activities of Daily Living | Care partners provide support to patients regarding completion of activities of daily living as needed |
| Medication | RNs help patients monitor their medication regimens to ensure safety and compliance |
| Labs | RNs order, request, and/or review patient labwork as needed |
